# Interventions to improve neurodevelopmental outcomes of children born moderate to late preterm: a systematic review protocol

**DOI:** 10.12688/gatesopenres.13246.2

**Published:** 2021-09-21

**Authors:** Josephine Agyeman-Duah, Stephen Kennedy, Frances O'Brien, Giancarlo Natalucci

**Affiliations:** 1 Nuffield Department of Women’s & Reproductive Health, University of Oxford, Oxford, UK; 2 Newborn Care Unit, John Radcliffe Hospital, Oxford University Hospitals NHS Foundation Trust, Oxford, UK; 3 Larsson-Rosenquist Foundation Center for Neurodevelopment, Growth and Nutrition of the Newborn, Department of Neonatology, University of Zurich and University Hopsital Zurich, Frauenklinikstrasse 10, CH-8091 Zürich, Switzerland

**Keywords:** Preterm, moderate preterm, late preterm, neurodevelopment, cognitive, neurobehaviour, motor development, intervention, outcome, parent, systematic review.

## Abstract

**Introduction: **Prematurity (birth before 37 ^+0^ weeks’ gestation) is associated with wide-ranging neurodevelopmental impairment. Prognosis among moderate to late (32 ^+0^ to <37 ^+0^  weeks’ gestation) preterm infants (MLPT) is better compared to their counterparts born very preterm (<32 ^+0^  weeks’ gestation). However the risk of developmental impairment among MLPT, who make up about 84% of all preterm infants, is 2-3 times higher when compared to infants born at term.

Early interventions have aimed to improve outcomes in preterm infants generally, but there are limited data on their need and effect in MLPT specifically. Prioritising research, long-term follow-up and early interventions targeted at ameliorating the impact of preterm birth among MLPT is required.

**Objectives: **To conduct a systematic review of the type of early childhood interventions (from birth until 4 years of age) offered to  MLPT children and to evaluate their impact on neurodevelopmental outcomes (cognitive, neurobehavioural and motor) as assessed in these children during childhood (until 18 years of age).

**Methods and analysis: **A systematic literature search in Web of Science, Medline Ovid, PsycINFO, CINAHL and EMBASE will be conducted. Data on MLPT children receiving developmental interventions until the age of 4 years will be evaluated. Interventions may involve parents or primary caregivers. Primary outcomes are cognitive, neurobehavioural and motor development as measured from birth until the age of 18 years.

The Cochrane Risk of Bias Assessment Tool will be used to evaluate the methodological quality of randomised controlled trials (RCTs) included in the review and will be graded as low, high  or unclear risk of bias. The quality of non-RCTs will be evaluated with the Newcastle-Ottawa Scale. ****The quality of evidence for each outcome will be evaluated using the Grading of Recommendations Assessment, Development and Evaluation Approach. Publication and reporting bias will be assessed using Egger’s test and funnel plots respectively.

## Introduction

### Outcomes of preterm birth on neurodevelopment

Preterm birth refers to any delivery that occurs before 37 ^+0^ weeks’ gestation. Preterm infants can be further defined as very, depending on their gestational age at birth, i.e. below 32 ^+0^, 32 ^+0^ to 33 ^+6^ and 34 ^+0^ to 36 ^+6^ weeks, respectively ^1^. Globally, 15 million babies are born preterm each year ^2^ and 84% are moderate to late preterm (MLPT). Due to advances in obstetrics and neonatal medicine, the survival rates of preterm babies overall have improved in the last few decades ^3, 4^. However, prematurity poses challenges for an individual’s neurodevelopmental outcome ^3^ from infancy into adulthood ^3^.

Developmental challenges span a spectrum of domains such as cognitive, motor, behavioural, vision, hearing, sleep and language skills ^5– 9^. The severity of developmental impairment is more pronounced with decreasing gestational age, i.e. the risk of developmental impairment among the very preterm is higher when compared with their MLPT counterparts ^4^.

### Risk of neurodevelopmental impairment among moderate to late preterm infants

Although the prognosis among MLPT is better compared to the very preterm, the risk of developmental impairment among MLPT, compared to their term counterparts is two-three times higher ^8, 10– 12^. During the last few months of pregnancy, the fetal brain increases rapidly in size and in complexity through intense neuronal branching, synaptogenesis and the beginning of myelination ^13, 14^. The premature exposure of the developing brain to an extrauterine environment is associated with an increased risk of neurodevelopmental impairment that manifests later as a cognitive, motor, behavioural or neurosensory deficit ^5, 15^.

### Interventions targeted at the neurodevelopment of moderate to late preterm infants

In addition to the biological risks associated with preterm birth, various environmental, social and demographic factors can adversely affect developmental outcomes. These factors include poor parent-infant relationships caused by parenting disturbance or parental stress, low socioeconomic status or parental education, and poor nurturing and infant malnutrition ^5, 16, 17^. Non-biological factors can also influence neurodevelopmental outcomes positively ^9, 18^ when properly coordinated. It is, therefore, important to consider interventions holistically when aiming to improve the neurodevelopmental outcomes of preterm infants. Therefore, this review will seek to identify all interventions that are targeted at improving cognitive, neurobehavioural and motor development of MLPT. 

Early childhood interventions (ECI) refer to the host of interventions delivered to an infant to address their development, including their physical, socio-emotional, cognitive and motor development beginning from birth until age eight ^19^ that aim to have significant and sustained impact on their development ^5, 20^. Studies have shown the wide-ranging benefits of ECI among preterm infants ^3, 9^. Many of these studies have focused on the developmental outcomes of very preterm infants ^15^. Little research has been conducted on MLPT with regards to their long-term follow-up, risk of developmental impairment, and available interventions to improve their neurodevelopmental outcomes ^12, 13^

Considering that MLPT constitute up to 84% of all preterm infants ^13^, any developmental impairment they experience places significant demand at the population level, related to demands on the health system, educational system and the economic implications for their families ^15, 21^. At the individual level, neurodevelopmental impairment may reduce the educational attainment of MLPT ^13^, which may further affect their economic power later in adulthood ^22^. It is, therefore, important for the health system to prioritise ECI targeted at ameliorating the impact of preterm birth among MLPT and for researchers to concentrate efforts in long-term follow-up of this preterm subgroup ^13^.

In a retrospective study by Kalia *et al.*, uptake of ECI services among MLPT was lower than among very preterm infants, except for MLPT with neonatal comorbidities ^23^. Therefore, enhancing parental awareness and education on the potential consequences of MLPT and the importance of early interventions for this cohort would almost certainly be useful.

### Previous studies

Similar to our systematic review, previous systematic reviews by Spittle *et al.* ^5^ reported on neurodevelopmental domains of cognition and motor development among all preterm subgroups (<37 ^+0^ weeks’ gestation, birth weight <2500 grams). They focused on RCT and quasi-RCT study designs and compared early intervention programmes with standard medical follow-up of preterm infants. Their study provides important findings on interventions that contribute to prevent cognitive and motor impairment among preterm infants. For example, they highlighted interventions such as a positive parent-infant relationship, physiotherapy, occupational therapy, infant stimulation, developmental care and early education. They further underscored the importance and positive influence of early interventions on the effects of prematurity and their impact in promoting cognitive and motor developmental outcomes among preterm infants.

Their review focused on all preterm infants but the authors also attempted a subgroup analysis of the effect of early interventions on neurodevelopmental outcomes by gestational age at birth. However, they found that most eligible studies in their review did not report outcomes according to gestational age, thus limiting subgroup analyses to ascertain the effects of early interventions among preterm subgroups. The few studies in their review which attempted a comparison by gestational age, did so mainly by comparing preterm infants born at <28 weeks’ gestation and ≥ 28 weeks’ gestation. This observation emphasises an important knowledge gap and the importance of our review, which aims to determine whether there are interventions that specifically target the neurodevelopment (cognitive, neurobehavioural and motor) of MLPT, a usually overlooked sub-population of preterm infants.

### Why it is important to do this review?

This systematic review will inform clinical practice in the field of ECI related to preterm birth, and help to improve neurodevelopmental outcomes for MLPT, a sizeable group that is mostly underrepresented in neonatal and preterm studies, but who may face significant developmental challenges.

**Review question:** Are there interventions to improve the neurodevelopmental (cognitive, neurobehavioural and motor) outcomes of MLPT?

## Objectives

In this systematic review, we aim to: a) identify which early interventions (from birth until 4 years of age) are being offered to MLPT children (32 ^+0^ to <37 ^+0^ weeks’ gestation) and b) evaluate the effects of those interventions on neurodevelopmental outcomes (cognitive, neurobehavioural and motor) up until 18 years of age.

## Protocol

This systematic review was registered in PROSPERO # CRD42021227749 (registered on 18 January 2021).

### Eligibility criteria

**Participants/population:** The population of interest in this systematic review include MLPT from birth until 18 years of age.

**Intervention(s), exposure(s):** Interventions considered in this systematic review for MLPT (including those involving parents or caregivers) may have been initiated in hospital but must thereafter have included, up until four years of age, at least one session in the community. Studies reporting on hospital or facility only interventions prior to hospital discharge will be excluded.

A predefined list of commonly occurring interventions that target cognitive, neurobehavioural and motor development have been considered.

a. Interventions for children:

•  Developmental therapy

•  Neurodevelopmental therapy

•  Educational therapy

•  Early intervention

•  Infant stimulation

•  Physical therapy

•  Occupational therapy

•  Speech therapy

•  Psychology

•  Exercise

•  Rehabilitation

b. Interventions for parent/caregiver which focus on the preterm child:

•  Parent education

•  Information given

•  Guided observation

•  Active involvement

c. Intervention programmes:

•  Avon Premature Infant Project

•  Curriculum and Monitoring System

•  Creating Opportunities for Parent Empowerment NICU Program

•  Infant Behavioural Assessment and Intervention Program

•  Infant Health and Development Program

•  Newborn Individualised Developmental & Assessment Programme

•  Neonatal Behavioural Assessment Scale, Brazelton

•  Nursing Systems for Effective Parenting-Preterm

•  Maternal Infant Transaction Program

•  Modified Mother Infant Transaction programme

•  Parent Baby Interaction Program

•  Parent Child Interaction Therapy

•  Premie Start

•  Sensory Integration and Neurodevelopmental Therapy

•  State Modulation

•  Victoria Infant Brain Studies

•  Supporting Play Exploration and Early Development Intervention

•  Traditional Holding

•  Parent-infant psychotherapy

**Comparator(s)/control**: Comparator groups are MLPT controls who receive no specific early interventions aimed at supporting their cognitive (including neurobehavioural) and motor development.

**Types of study to be included:** This systematic review will include analytical studies (such as retrospective cohort, prospective cohort, case-control studies); observational studies and experimental studies (such as RCTs and quasi-experimental designs). Studies reported in conference abstracts and other forms of grey literature utilising the above study designs with high evidence of data will be included in this review. Language restrictions will not be applied. Multiple reports of the same sample will be treated as a single study.

**Exclusion:** Studies that focus on very preterm infants and animal studies will be excluded. In terms of study design, studies with low evidence of data such as case series, cross-sectional studies, case reports, editorials, commentaries, qualitative studies and literature reviews will be excluded.

**Information sources:** Electronic databases including Web of Science, Medline Ovid, PsycINFO, CINAHL and EMBASE will be searched. The reference lists of papers identified through the database searches will be scanned to identify further studies of relevance to this systematic review. Clinical trials registers such as the ClinicalTrials.gov: www.clinicaltrials.gov, Cochrane Central Register of Controlled Trials (CENTRAL): www.cochranelibrary.com and the World Health Organization International Clinical Trials Registry Platform (WHO ICTRP): www.who.int/ictrp will be consulted for additional relevant studies.

**Search strategy**: The search strategy includes only terms relating to or describing the population, interventions, comparator and outcomes of interest. The search terms have been adapted for use with the different bibliographic databases in combination with database-specific filters for clinical trials, RCTs, systematic reviews, meta-analyses and in combination with externally validated search terms for observational studies. An expert librarian was consulted in refining the search terms (please see https://doi.org/10.6084/m9.figshare.14494794 for Medline search strategy).

Searches will include all published studies from inception of the databases until the date the searches are run. The searches will be re-run just before the final analyses and further studies retrieved for inclusion. Our initial search strategy yielded 5,262 results from the five databases we searched.

**Main outcome(s):** The primary outcome of interest will be components of interventions that contribute to MLPT cognitive, neurobehavioural and motor development.

**Outcome measures:** All domains of cognitive, neurobehavioural and motor outcomes as measured by any assessment tool will be considered.

**Additional outcome(s):** The effectiveness of interventions that promote cognitive (including language development score), motor and neurobehavioural (including socioemotional development) among MLPT.

**Measures of effect:** The impact of early interventions for the promotion of cognitive, neurobehavioural and motor outcomes will be reported as relative risks and mean differences.

**Data extraction (selection and coding):** A combination of appropriate keywords from the study’s keywords (for the population, interventions, comparator and outcomes, PICO) has been adapted with relevant truncations and BOOLEAN operators to suit each database search.

**Data management:** All records sourced from the database searches will be saved in Endnote Library (EndNote X9, Clarivate, Boston MA, USA). Rayyan software ^1^ will be used for title and abstract screening. A data extraction sheet has been created to collate relevant information from studies for study quality and evidence synthesis. Data synthesis will be performed using RevMan 5.1 software (The Cochrane Collaboration, London, UK).

**Data collection process**: A data collection template has been designed to extract data from reports. This includes headings such as the study setting, population characteristics (for example the age groups and gestational ages of the population of interest), type of study, interventions, neurodevelopmental outcomes and information for assessment of the risk of bias. The data template will be piloted independently before the main data extraction commences. 

Two primary reviewers will extract data independently; discrepancies will be resolved through discussion. A third reviewer will be involved where necessary. Authors of included studies will be contacted for any relevant or missing data, where needed.

**Selection process:** Two primary reviewers will independently screen the records identified through the database searches in a three-step process: firstly, to remove duplicate studies, secondly to conduct title and abstract screening to remove irrelevant records based on the study’s inclusion and exclusion criteria, and thirdly to read thoroughly through all relevant records from step two to identify studies to be included in the data and statistical analysis. The reviewers will discuss any discrepancies in the selected studies and if another opinion is needed, a third reviewer with expert subject matter knowledge will be consulted to provide clarity. The reference list from all relevant articles to be included in the review will be examined for additional studies of interest, which may have been missed in the database searches.

**Risk of bias (quality) assessment:** The Cochrane Risk of Bias Assessment Tool will be used to evaluate the methodological quality of RCTs included in the review and graded as low-risk, high-risk or unclear risk of bias. The quality of non-RCTs will be evaluated with the Newcastle-Ottawa Scale.

**Confidence in cumulative evidence**: The quality of evidence for each outcome will be evaluated using the Grading of Recommendations Assessment, Development, and Evaluation (GRADE) approach.

**Meta-bias(es):** Publication bias will be assessed using Egger’s test and reporting bias will be evaluated using Funnel plots.

Two primary reviewers will independently assess the risk of bias in included studies for: sequence generation, allocation concealment, blinding, incomplete outcome data, selective outcome reporting and any other source of potential bias. The two reviewers will resolve any disagreements in the risk of bias assessment through thorough discussion. A third reviewer will be involved to resolve disagreements where necessary.

**Strategy for data synthesis:** Two independent reviewers will be involved at each step of the review, starting from the records identification stage. A third reviewer will be consulted to provide clarity where needed. Content analysis will be conducted through an iterative process and a qualitative description of information extracted from all relevant studies will be coded for interpretation.

The heterogeneity of included studies will be assessed using chi squared test (significant level 0.1) and I-squared statistics. Homogeneity will be categorised according to PRISMA-P classification ^24^: 0 to 40% as not important heterogeneity, 30 to 60% as moderate heterogeneity, 50 to 90% as substantial heterogeneity and 75 to 100% as considerable heterogeneity. Subgroup analysis will be performed to explore substantial heterogeneity (of >50%). Where the heterogeneity is not significant, a fixed effect with the Mantel-Haenszel method will be used for the data synthesis ^25^.

Data synthesis will only be narrative where the heterogeneity is considerable (i.e. χ ^2^, p > 0.1 and I ^2^ ≥ 50%). A narrative synthesis will include the type of intervention, target population characteristics, type of outcome and interventions. Risk ratios will be calculated for dichotomous outcomes and standardised mean difference (at 95% confidence interval) for continuous outcomes.

Where heterogeneity may be due to chance, (i.e. where studies have used the same type of intervention and comparator with the same outcome measure) random-effects meta-analysis may be performed with standardised mean differences for continuous outcomes and risk ratios for binary outcomes at 95% confidence interval and two-sided P values for each outcome. In studies where the effects of clustering have not been considered, we will adjust the standard deviations for the design effect.

A meta-analysis will be performed as a follow-up study if the data extracted are found to be sufficiently homogenous.

**Analysis of subgroups or subsets:** Analysis of MLPT subgroups will be performed on characteristics such as gestational age at birth, age at intervention and the characterisation of interventions (such as type, dose, frequency, duration of intervention and person delivering the intervention) if sufficient data are retrieved.

**Type and method of review:** Systematic review. A meta-analysis will be performed if the included studies are sufficiently homogeneous.

Ethics approval is not required for this systematic review. The secondary data to be analysed will be data from participants enrolled in studies or trials for which ethical approval has been sought. Findings from the review will be published in a peer-reviewed journal, presented at conferences and meetings and widely disseminated through academic and neonatal professional and parent support group channels.

## Study status

The search strategies and data extraction tools have been developed; data collection and analysis will be completed by autumn 2021.

## Data availability

### Underlying data

No data are associated with this article.

### Reporting guidelines

Figshare: PRISMA-P checklist and the search strategy for ‘Interventions to improve neurodevelopmental outcomes of children born moderate to late preterm: a systematic review protocol’, https://doi.org/10.6084/m9.figshare.14494794 ^26^

Data are available under the terms of the Creative Commons Zero "No rights reserved" data waiver (CC BY 4.0 Public domain dedication).
