# Supplementary material for: Interventions to improve neurodevelopmental outcomes of children born moderate to late preterm: a systematic review protocol
Source: Gates Open Res. 2021 Sep 21;5:78. Originally published 2021 May 12. [Version 2] doi: 10.12688/gatesopenres.13246.2 (PMC8369897; doi:10.12688/gatesopenres.13246.2)
Supplement: Peer review history

# Peer review: Reviewer response for version 1

**DOI:** 10.21956/gatesopenres.14480.r30857

**Published:** 2021-08-16

**Authors:** Hilary Wong

**Affiliations:** 1 Department of Paediatrics, University of Cambridge, Cambridge, UK

The authors described their protocol for a comprehensive systematic review on early childhood interventions to improve the neurodevelopmental outcomes in infants born moderate to late preterm. The protocol is well-written, providing a clear background to this important and yet under-researched area. The planned methods are comprehensively described and covered all the required elements of a good systematic review. I only have a few minor points:

 1. Search strategy:

 The authors present a link to the search strategy developed. It will useful to get a sense of how sensitive or precise this preliminary search was i.e. how many articles were returned.

 2. Outcome measures:

 The authors state that they intend to include all outcomes relating to cognition, neurobehavioural and motor domains. It will be beneficial to know the potential impairments that have been reported in the MLPT population, covered under these umbrella terms. Perhaps the authors can expand further. For example, attention deficit or language impairment are described but not included in the search terms in the search strategy.

 3. As described by the authors in the section 'previous studies', there will be intervention studies that included all preterms but perhaps not report results according to gestational age groups. Is there any intention to seek data on the MLPT sub-group of interest directly from researchers to include in this systematic review?

Is the study design appropriate for the research question?

Yes

Is the rationale for, and objectives of, the study clearly described?

Yes

Are sufficient details of the methods provided to allow replication by others?

Yes

Are the datasets clearly presented in a useable and accessible format?

Not applicable

Reviewer Expertise:

Neonatology, neurodevelopmental follow-up, perinatal epidemiology, genetics of preterm birth and developmental outcomes

I confirm that I have read this submission and believe that I have an appropriate level of expertise to confirm that it is of an acceptable scientific standard.

# Response

**Published:** 2021-09-06

**Authors:** Josephine Agyeman-Duah

**Affiliations:** University of Oxford, UK

The authors described their protocol for a comprehensive systematic review on early childhood interventions to improve the neurodevelopmental outcomes in infants born moderate to late preterm. The protocol is well-written, providing a clear background to this important and yet under-researched area. The planned methods are comprehensively described and covered all the required elements of a good systematic review. 

 I only have a few minor points:

**Comment 1:** Search strategy:

 The authors present a link to the search strategy developed. It will useful to get a sense of how sensitive or precise this preliminary search was i.e. how many articles were returned.

**Reply 1:** Thank you. We have indicated the number of results the search strategy returned. Please see page 8. 

**Comment 2:** Outcome measures:

 The authors state that they intend to include all outcomes relating to cognition, neurobehavioural and motor domains. It will be beneficial to know the potential impairments that have been reported in the MLPT population, covered under these umbrella terms. Perhaps the authors can expand further. For example, attention deficit or language impairment are described but not included in the search terms in the search strategy.

**Reply 2:** Thank you. We will consider all relevant interventions that target the three neurodevelopmental domains: cognitive, motor and neurobehaviour. We have provided examples under the additional outcomes to reflect language and socioemotional development.

**Comment 3:** As described by the authors in the section 'previous studies', there will be intervention studies that included all preterms but perhaps not report results according to gestational age groups. Is there any intention to seek data on the MLPT sub-group of interest directly from researchers to include in this systematic review?

**Reply 3:** Yes. We will contact authors for missing data as well as data for the subgroups where studies report results for preterm infants of all gestational age groups. Thank you.

# Peer review: Reviewer response for version 1

**DOI:** 10.21956/gatesopenres.14480.r30820

**Published:** 2021-08-11

**Authors:** Elaine M. Boyle

**Affiliations:** 1 Department of Health Sciences, University of Leicester, Leicester, UK

This paper presents a proposal for a systematic review on interventions to improve neurodevelopmental outcomes of children born at moderately preterm (32-33 weeks of gestation)-late preterm (34-36 weeks of gestation). This is an area that has been under-researched to date, given that later difficulties in this population have only been recognised relatively recently. This systematic review is therefore timely and useful.

 The authors have clearly outlined and justified their case, with appropriate references and context. The methodology to be used is appropriate and uses standard and accepted systematic review methods. The search strategy is clear and appropriate. I think that the results of this review will be helpful to guide future research in this area.

 I have some minor comments on the paper that I would like to see addressed:

1. The authors have chosen to include both moderately preterm and late preterm birth in this review, and group them together. I think that this paper should include a definition of each of these gestational age groups, for clarity, as these are two distinct groups of infants, with somewhat different characteristics. Neonatal morbidities are generally different in the moderately preterm than the late preterm infants. Similarly, the focus of care is different, with moderately preterm infants routinely admitted to a neonatal unit after birth. There may also be differences with respect to post-discharge follow-up/surveillance. Whilst this is not directly relevant to later interventions, I think it would be helpful to see justification for the amalgamation of two slightly different populations.
2. Throughout the paper, preterm infants are referred to as "preterms". A minor detail, but "preterm" is an adjective, and "preterm baby/infant" is more correct.
3. In the exclusion criteria, the term "very early preterm" is not correct - I think it should read "very preterm".

.

Is the study design appropriate for the research question?

Yes

Is the rationale for, and objectives of, the study clearly described?

Yes

Are sufficient details of the methods provided to allow replication by others?

Yes

Are the datasets clearly presented in a useable and accessible format?

Not applicable

Reviewer Expertise:

Outcomes of moderately preterm, late preterm and early term birth; Neonatal pain.

I confirm that I have read this submission and believe that I have an appropriate level of expertise to confirm that it is of an acceptable scientific standard.

# Response

**Published:** 2021-09-06

**Authors:** Josephine Agyeman-Duah

**Affiliations:** University of Oxford, UK

**Reviewer 1:**

 This paper presents a proposal for a systematic review on interventions to improve neurodevelopmental outcomes of children born at moderately preterm (32-33 weeks of gestation)-late preterm (34-36 weeks of gestation). This is an area that has been under-researched to date, given that later difficulties in this population have only been recognised relatively recently. This systematic review is therefore timely and useful.

 The authors have clearly outlined and justified their case, with appropriate references and context. The methodology to be used is appropriate and uses standard and accepted systematic review methods. The search strategy is clear and appropriate. I think that the results of this review will be helpful to guide future research in this area.

 I have some minor comments on the paper that I would like to see addressed:

**Comment 1:** The authors have chosen to include both moderately preterm and late preterm birth in this review, and group them together. I think that this paper should include a definition of each of these gestational age groups, for clarity, as these are two distinct groups of infants, with somewhat different characteristics.

**Reply 1:** Thank you. We have classified moderate and late preterm birth as distinct categories. Please see pages 3 and 4.

**Comment 2:** Neonatal morbidities are generally different in the moderately preterm than the late preterm infants. Similarly, the focus of care is different, with moderately preterm infants routinely admitted to a neonatal unit after birth. There may also be differences with respect to post-discharge follow-up/surveillance. Whilst this is not directly relevant to later interventions, I think it would be helpful to see justification for the amalgamation of two slightly different populations.

**Reply 2:** Thank you. Our study intends to describe the interventions and outcomes between the two subgroups as distinct populations. Therefore, we have made the changes on pages 3 and 4 to reflect the subgroups as distinct moderate and late preterm infant groups. Please see pages 3 and 4.

**Comment 3:** Throughout the paper, preterm infants are referred to as "preterms". A minor detail, but "preterm" is an adjective, and "preterm baby/infant" is more correct.

**Reply 3:** Thank you. The term ‘preterms’ has been corrected to read as ‘preterm infants’.

**Comment 4:** In the exclusion criteria, the term "very early preterm" is not correct - I think it should read "very preterm".

**Reply 4:** Thank you. We have corrected the text accordingly. Please see page 8.
